# Clinical significance of reactive thrombocytosis in the course of acute pancreatitis

**DOI:** 10.1186/s12876-023-02837-w

**Published:** 2023-06-13

**Authors:** Nobutaka Chiba, Atsunori Sugita, Minori Mizuochi, Jun Sato, Takeshi Saito, Atsushi Sakurai, Kosaku Kinoshita

**Affiliations:** Division of Emergency and Critical Care Medicine, Department of Acute Medicine, Nihon University School of Medicine, 30-1 Oyaguchi-kamimachi, Itabashi-Ku, Tokyo, 173-8610 Japan

**Keywords:** Thrombocytosis, Severe acute pancreatitis, Pancreatic complications, Platelet activation, Inflammation

## Abstract

**Background:**

Reactive thrombocytosis occurs secondary to systemic infections, inflammatory, and other conditions. The relationship between thrombocytosis and acute pancreatitis (AP) in inflammatory diseases is uncertain. This study aimed to evaluate the clinical significance of thrombocytosis in AP patients during hospitalization.

**Methods:**

Subjects within 48 h of AP onset were consecutively enrolled over 6 years. Platelet counts of ≥ 450,000/µL were defined as thrombocytosis, < 100,000/µL as thrombocytopenia, and other counts as normal. We compared clinical characteristics, including the rate of severe AP (SAP) assessed by the Japanese Severity Score; blood markers, including hematologic and inflammatory factors and pancreatic enzymes during hospitalization; and pancreatic complications and outcomes in the three groups.

**Results:**

A total of 108 patients were enrolled. Although, SAP was more common in patients with thrombocytosis and thrombocytopenia (87.9% and 100%, respectively), the differences in lymphocytes and C-reactive protein, lactase dehydrogenase, and antithrombin levels, which are factors of the systemic inflammatory response, and the mean platelet volume, an indicator of platelet activation, were observed among patients with thrombocytosis and thrombocytopenia during hospitalization. Regarding pancreatic complications and outcomes, patients with thrombocytosis and thrombocytopenia had higher acute necrotic collection (ANC), pancreatic necrosis, intestinal paralysis, respiratory dysfunction, and pancreatic-related infection levels than patients with normal platelet levels. The relationship between pancreatic complications and thrombocytosis was assessed by multivariate logistic regression; the odds ratios for development of ANC, pancreatic necrosis and pancreatic-related infections were 7.360, 3.735 and 9.815, respectively.

**Conclusions:**

Thrombocytosis during hospitalization for AP suggests development of local pancreatic complications and pancreatic-related infections.

## Introduction

Acute pancreatitis (AP) is not simply a local inflammatory disease limited to pancreatic and peripancreatic tissue; it can present in its most serious form as systemic inflammatory response syndrome (SIRS), multiple organ failure, sepsis, and death [1]. The disease is self-limiting and mild in the majority of patients, but in up to 25% of patients, the disease is severe due to local and systemic complications. Systemic complications such as pulmonary, renal, and cardiocirculatory insufficiency, coagulopathy, and sepsis occur in 20-80% of patients with severe necrotizing pancreatitis (SNP) [2–5]. In general, organ dysfunction due to systemic inflammation is caused by the release of tumor necrosis factor-ɑ (TNF-ɑ) and other proinflammatory mediators into the circulation [6]. Increased levels of TNF-ɑ, interleukin-1 (IL-1), and interleukin-6 (IL-6) have also been observed during the onset and progression of AP [7, 8]. In addition, the involvement of these mediators has been reported along with abnormal levels of factors involved in systematic inflammation, such as C-reactive protein (CRP) [9], lactate dehydrogenase (LD) [10] and antithrombin (AT) [11], and a decrease in peripheral lymphocytes caused by apoptosis has also been reported to cause a decrease in cellular immunity. These markers have been shown to vary with the severity of AP and to be correlated with the incidence of systemic infections in the early stages of AP [12].

In addition to their well-known functions of hemostasis and thrombosis, platelets have been reported to play a proinflammatory role [13]. The enhancement of inflammation by platelet activation has been previously described in models of systemic inflammation, such as ulcerative colitis and Crohn’s disease [14]. In particular, the mean platelet volume (MPV), a parameter of platelet activation, has been shown to reflect the inflammatory burden and disease activity in inflammatory diseases, such as gastrointestinal diseases with inflammation [15] and rheumatoid arthritis [16]; metabolic diseases, such as diabetes [17], obesity [18] and starvation [19]; infection [20]; thyroid diseases [21]; and cardiovascular diseases [22]. Similarly, tissue damage and the release of inflammatory mediators resulting from the onset and progression of AP have been reported to result in platelet activation [23] and influence MPV [24]. These reactions consume platelets, but in less severe cases, the platelet count is maintained at a constant level by hyperplasia in the bone marrow [23]. In more severe cases, platelet count is shown to be decreased and correlates negatively with CRP concentration [25]. On the other hand, it is known that a pro-inflammatory state mediated by cytokines, particularly promotion of thrombus formation by IL-6, can lead to reactive platelet proliferation [26]. Reactive thrombocytosis occurs secondary to systemic infections, inflammatory conditions, bleeding, iron deficiency anemia and tumors [27], and reactive thrombocytosis in SAP has been reported [25]. However, there are few clinical reports of this process, which causes systemic inflammation, in AP [28, 29]. The prevalence, characteristics, timing and impact of thrombocytosis in AP are not well known. Therefore, the aim of this study was to evaluate the clinical significance of reactive thrombocytosis during hospitalization for AP.

## Methods

### Study design and patients

This study was approved by the Clinical Research Review Committee of Nihon University Hospital (No. 161,202) and was designed as a single-institution prospective observational investigation using the database of patients treated for AP admitted to our hospital from January 2015, through December 2020. In this study, demographic and clinical characteristics were reviewed from patients’ electronic medical records.

The diagnostic criteria for AP are based on two or more of the following conditions: (1) upper abdominal pain of acute onset, (2) serum amylase or lipase activity three times greater than normal, and (3) finding on cross-sectional abdominal imaging consistent with AP [28]. To ensure eligible patients, those with pancreatic tumors, immune diseases and admission to the hospital more than 48 h after the onset of symptoms or incomplete laboratory examination results were excluded. All blood samples were collected within 2 h after admission and analyzed within 6 h at the same laboratory in our hospital. After hospitalization, blood samples were collected daily for up to 14 days and at least once every 2 days until resolution of symptoms, oral intake, and normalization of pancreatic enzymes. Complete blood count (CBC) analysis was performed with the same analyzer within 2 h after collection of blood samples with the use of a Beckman Coulter (High Wycombe, UK) UniCel DxH 800 Coulter Cellular Analysis System.

The severity of disease was determined by the criteria of the Japanese Ministry of Health, Labor and Welfare study group for acute pancreatitis severity (2008) (Japanese Severity Score [JSS]) (Table 1) for early diagnosis and appropriate treatment; SAP was diagnosed when the total prognostic factor score was 3 points or more or the computed tomography (CT) grade was 2 or more [30]. We evaluated prognostic factor scores and/or contrast-enhanced computed tomography (CECT) grades within 48 h after admission because the JSS recommends repeating severity assessments in the first 48 h. In addition, at least one CT scan was performed within 14 days. Computed tomography scans were evaluated by a radiologist.

**Table 1:** The Severity Scoring System for Acute Pancreatitis of the Japanese Ministry of Health, Labor and Welfare (2008)

Severe acute pancreatitis: prognostic factor ≥ 3 or CT grade ≥ 2		
Prognostic Factors (1 point for each factor)		
1. Base excess ≤ -3 mEq/L or shock (systolic blood pressure < 80 mm Hg) 2. PaO2 ≤ 60 mm Hg (room air) or ventilatory failure (ventilator management is needed) 3. BUN ≥ 40 mg/dL (or Cr ≥ 2.0 mg/dL) or oliguria (daily urine output < 400 mL even after IV fluid resuscitation) 4. LDH ≥ 2 times the upper limit of normal 5. Platelet count ≤ 100,000/mm^3^ 6. Serum Ca ≤ 7.5 mg/dL 7. CRP ≥ 15 mg/dL 8. Number of positive measures in SIRS criteria ≥ 3 9. Age ≥ 70 y		
**CT Grade By Contrast-Enhanced CT**		
1. Extrapancreatic progression of inflammation		
Anterior pararenal space		0 point
Root of mesocolon		1 point
Beyond lower pole of kidney		2 points
2. Hypoenhanced lesion of the pancreas		
The pancreas is conveniently divided into 3 segments (head, body, and tail).		
Localized to one segment or surrounding the pancreas	0 point	
Covers 2 segments	1 point	
Occupies 2 complete segments or more	2 points	
1 + 2 = total score		
Total score = 0 or 1		Grade 1
Total score = 2		Grade 2
Total score = 3 or more		Grade 3

Measures included in SIRS diagnostic criteria: (1) temperature, > 38°C or < 36°C; (2) heart rate, > 90 beats/min; (3) ventilatory rate, > 20 breaths/min or PaCO2 < 32 torr; (4) WBC, > 12,000 cells/mm^3^, < 4000 cells/mm^3^, or > 10% immature (band) forms

BUN, blood urea nitrogen; IV, intravenous; CRP, C-reactive protein; LDH, lactate dehydrogenase; PaO_2_, partial pressure of oxygen in blood; SIRS, systemic inflammatory response syndrome; WBC, white blood cell

Data, including the patient age and sex, the etiology of AP and the following prognostic scores were collected within 48 h after admission [31–34]: (1) Acute Physiology and Chronic Health Evaluation II (APACHE II) score; (2) Sequential Organ Failure Assessment (SOFA) score; (3) Modified CT Severity Index (MCTSI); and (4) Bedside Index for Severity in Acute Pancreatitis (BISAP) score. The severity grade under the revised Atlanta classification [35] was used to evaluate local or systemic complications and was assessed more than 48 h after admission. CBC parameters and acute physiological parameters were collected daily for up to 14 days and at least once every 2 days.

Postdiagnosis treatments included intravenous fluid administration within 24 h to achieve a mean arterial pressure of 65 mmHg or higher and a urine output of 0.5 ml/kg/h. Analgesics were administered when necessary. If SAP was diagnosed, patients were administered enteral nutrition (EN) within 48 h, dialysis due to renal failure, mechanical ventilation, prophylactic antibiotics, and protease inhibitors.

### Outcome measurements

We examined the variation in the highest and lowest platelet counts from admission to discharge and classified the patients into three groups: the thrombocytopenia group was defined as those with platelet counts of less than 100,000/µL for two or more consecutive days, the thrombocytosis group as those with platelet counts of 450,000/µL or more, and the normal platelet count group as the remaining patients [27]. Patients in the thrombocytopenia group whose platelet counts increased during hospitalization were considered to be in the thrombocytopenia group.

We compared the incidence of systemic or local pancreatic complications and outcomes in these three groups. As a secondary analysis, we evaluated the incidence of pancreatic complications and outcome in the thrombocytosis group. We evaluated systemic complications, including respiratory, renal, and cardiovascular dysfunction. Respiratory dysfunction was defined as PaO2/FiO2 less than 300 lasting more than 48 h, renal dysfunction was defined as creatinine more than 1.9 mg/dL, and cardiovascular dysfunction was defined as nonresponsiveness to infusion, systolic blood pressure less than 90 mmHg, or the use of vasopressors [35]. Local complications were defined as the incidence of acute peripancreatic fluid collection (APFC), acute necrotic collection (ANC), and pancreatic necrosis. Intestinal paralysis was defined as gastric outlet dysfunction and ileus, including intestinal necrosis and abdominal compartment syndrome. Vascular complications were defined as the occurrence of pseudoaneurysm and venous thrombosis. The occurrence of local pancreatic complications was confirmed with CT images. The outcome was evaluated as the rate of pancreatic-related infection, mortality within 14 days, and total mortality. We defined pancreatic-related infection as the presence of bacteria based on blood culture or local culture obtained by percutaneous, image-guided, or endoscopic fine-needle aspiration or the presence of extraluminal gas in the pancreatic and/or peripancreatic tissue in contrast-enhanced CT [30].

### Statistical analysis

Statistical Production and Services Solution 22.0 (SPSS 22.0, SPSS Inc., Chicago IL, USA) was used for statistical analysis. The distribution of variables in the study groups was analyzed by the Kolmogorov-Smirnov test. Normally distributed variables were compared by the t-test, and the results are expressed as the mean ± standard deviation (SD). Variables without a normal distribution were compared with the Mann‑Whitney U-test; the results are expressed as the median (interquartile range). Qualitative variables are expressed as numbers and percentages. A *p* value < 0.05 was set as the definition of statistical significance.

Differences between the two groups, mild and severe AP, were analyzed by Student’s t test for normally distributed continuous variables and the Mann–Whitney U test for variables without a normal distribution; the χ2 test was used for categorical variables. Physiological data, systemic or local complications, and outcomes from each platelet group were compared by one-way analysis of variance (ANOVA). Subsequently, the Tukey–Kramer test was performed. We also performed multivariable logistic regression analysis to evaluate the effect of thrombocytosis on systemic and local complications [35] and outcomes in patients with AP.

## Results

A total of 121 patients with AP were enrolled in the study. Of those, thirteen patients were excluded from the analysis; five patients were admitted to the hospital more than 72 h after the onset of symptoms. Three patients had tumors, and two patients had immune disease. Three patients had incomplete laboratory examination results. The study population accordingly consisted of 108 patients: 33 patients (30.6%) with thrombocytosis, 24 patients (22.2%) with thrombocytopenia, and 51 patients (47.2%) with normal values. The peak platelet count of the thrombocytosis group was 590,000 ± 144,000/µL, the lowest value of the thrombocytopenia group was 63,100 ± 24,500/µL, and the highest value of the normal value group was 290,000 ± 69,300/µL, and the number of days from the onset of pancreatitis was 12.22 ± 3.03 days, 3.17 ± 1.68 days and 7.94 ± 2.64 days, respectively.

Table 2 shows the demographic and clinical characteristics of this study when the patients were divided into two groups: a mild group and a severe group based on the JSS. Significantly higher APACHE II score, SOFA score, and SIRS score values; higher WBC, neutrophil count, LD and CRP values; and lower platelet count and calcium levels were observed in the SAP group. In the SAP group, there were 26 (51%) patients in the normal value group, 29 (87.9%) patients in the thrombocytosis group, and 24 (100%) patients in the thrombocytopenia group.

**Table 2:** Comparison of mild and severe pancreatitis based on the JSS

	All (n = 108)	Mild (n = 29)	Severe (n = 79)	*p* value
(A) Characteristics				
Age	54.5 (17.2)	54.3 (14.8)	54.6 (18.1)	0.949
Sex (male), n (%)	82 (75.9)	24 (82.8)	58 (73.4)	0.447
Etiologic factor, n (%)				0.552
Alcohol use	55 (50.9)	12 (41.4)	43 (54.4)	
Gallstones	18 (16.7)	7 (24.1)	11 (13.9)	
Idiopathic	17 (15.7)	5 (17.2)	12 (15.2)	
Other cause	18 (16.7)	5 (17.2)	13 (16.5)	
APACHE II score, mean (SD)	9.09 (5.79)	5.45 (3.05)	10.43 (5.98)	< 0.001
SOFA score, median (IQR)	2 (1–4)	0 (0–2)	2 (1–4)	< 0.001
SIRS score, median (IQR)	2 (1–3)	1 (0-1.5)	3 (1–3)	< 0.001
WBC (×10^3^/µL)	12.8 (5.67)	10.7 (3.42)	13.5 (6.13)	0.020
Neutrophil (×10^3^/µL)	10.9 (5.51)	8.92 (3.36)	11.6 (5.95)	0.024
Lymphocyte (×10^3^/µL)	1.0 (0.59)	0.98 (0.63)	1.08 (0.5)	0.441
RDW	13.9 (1.53)	13.9 (1.01)	13.9 (1.69)	0.929
Platelet (×10^3^/µL)	198.98 (81.26)	254.1 (70.7)	178.1 (75.5)	< 0.001
MPV	8.33 (1.07)	8.23 (0.66)	8.37 (1.18)	0.560
BUN (mg/dl)	17.6 (16.7)	15.9 (14.7)	18.1 (17.4)	0.544
Creatinine (mg/dl)	0.92 (0.75)	0.84 (0.5)	0.96 (0.83)	0.460
LD (U/L)	375.2 (245.2)	237.2 (66.9)	426.6 (267.0)	< 0.001
Ca (mg/dL)	8.13 (1.25)	8.98 (0.45)	7.86 (1.30)	< 0.001
CRP (mg/dl)	13.5 (14.2)	5.34 (8.83)	16.5 (14.6)	< 0.001
(B) Platelet count groups, n (%)				< 0.001
Normal value	51 (47.2)	25 (49.0)	26 (51.0)	
Thrombocytosis	33 (30.6)	4 (12.1)	29 (87.9)	
Thrombopenia	24 (22.2)	0 (0)	24 (100)	

JSS: Japanese Severity Score, APACHE II: Acute Physiology and Chronic Health Evaluation II, SOFA: Sequential Organ Failure Assessment, SIRS: Systemic inflammatory response syndrome, WBC: White blood cell, RDW: Red blood cell volume distribution width, MPV: Mean platelet volume, LD: Lactase dehydrogenase, CRP: C-reactive protein

Clinical characteristics of patients in the three groups are shown in Table 3. In the severity assessment, APACHE II scores, prognostic factors, BISAP scores, the revised Atlanta Classification and MCTSI scores were significantly different among the three groups. APACHE II scores, prognostic factors, and MCTSI scores increased stepwise in the normal value, thrombocytosis, and thrombocytopenia groups.

**Table 3:** Clinical characteristics of patients in different platelet count groups

	Normal value	Thrombocytosis	Thrombopenia	*p* value
Cases: numbers	51	33	24	
(A) Baseline characteristics				
Age	56.8 (17.3)	50.9 (17.6)	50.9 (17.6)	0.312
Sex (male), n (%)	38 (74.5)	27 (81.8)	17 (70.8)	0.600
Etiologic factor, n (%)				0.273
Alcohol use	23 (45.1)	16 (48.5)	16 (66.7)	
Gallstones	9 (17.9)	4 (12.1)	5 (20.8)	
Idiopathic	11 (21.6)	5 (15.2))	1 (4.2)	
Other cause	8 (15.7)	8 (24.2)	2 (8.3)	
(B) Severity assessment				
APACHE II score, mean (SD)	6.78 (4.31)	8.76 (5.00)	14.46 (6.21)	< 0.001
Prognostic factor, median (IQR)	1 (0–2)	2 (1–3)	4 (3–5)	< 0.001
BISAP, median (IQR)	1 (0–2)	2 (1–3)	2 (1–3)	< 0.001
Revised Atlanta classification, n (%)				< 0.001
Mild	23 (45.1)	3 (9.1)	1 (4.2)	
Moderate	25 (49.0)	17 (51.5)	9 (37.5)	
Severe	3 (5.9)	13 (39.4)	14 (58.3)	
MCTSI, median (IQR)	2 (2–6)	4 (2–6)	6 (4–10)	0.025

APACHE II: Acute Physiology and Chronic Health Evaluation II, BISAP: Bedside Index for Severity in Acute Pancreatitis, MCTSI: Modified CT Severity Index

A. Hematologic factors

**Fig. 1:**
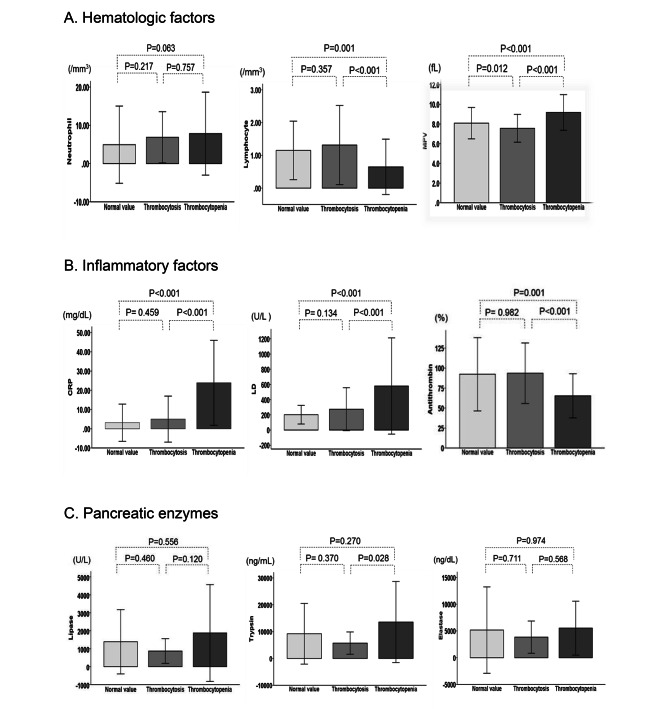
shows hematologic factors, including neutrophils, lymphocytes and MPV (Fig. 1.A); inflammatory factors, including CRP, LDH and antithrombin (Fig. 1.B); and pancreatic enzymes, including lipase, trypsin and elastase (Fig. 1.C) among each platelet group. In the thrombocytosis group, MPV, RDW, CRP, LD, and trypsin were decreased compared to those in the thrombocytopenia group. Lymphocyte and AT levels were increased MPV: Mean platelet volume, LD: Lactase dehydrogenase, CRP: C-reactive protein A. Systemic complications

Figure 1 shows the results of the assessment of hematologic factors, including neutrophils, lymphocytes and MPV (Fig. 1.A); inflammatory factors, including CRP, LD and AT (Fig. 1.B); and pancreatic enzymes, including lipase, trypsin and elastase (Fig. 1.C), on the days when the platelet levels fluctuated in each group. In the thrombocytosis group and normal platelet count group, MPV, CRP, and LD levels were lower than those in the thrombocytopenia group. Lymphocyte and AT levels were higher. MPV was significantly lower in the thrombocytosis group than in the normal and thrombocytopenia groups. On the other hand, there was no significant difference in neutrophils, lipase or elastase among the three groups.

**Fig. 2:**
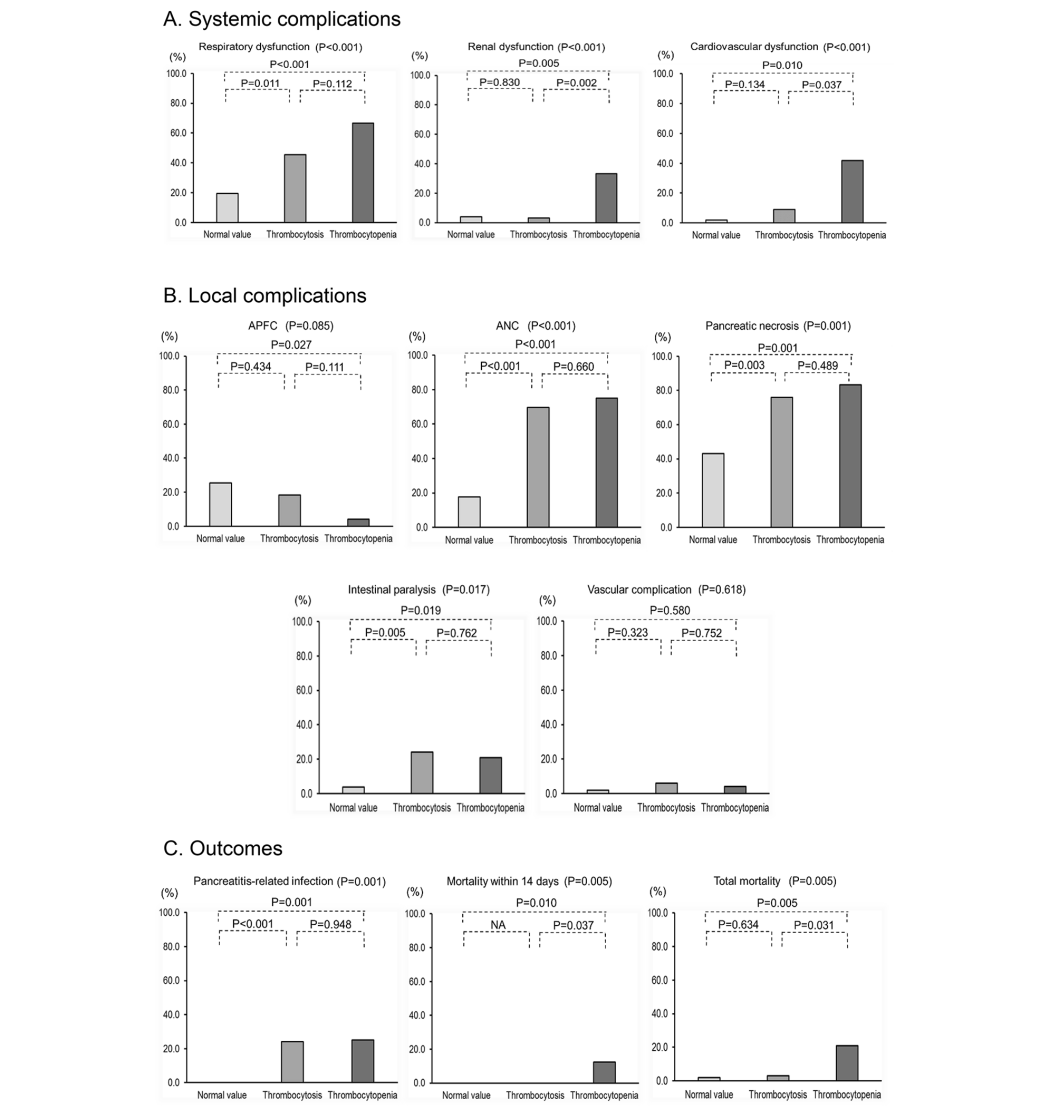
shows the systemic complications, including respiratory, renal and cardiovascular dysfunction (Fig. 2.A); local complications, including APFC, ANC, pancreatic necrosis, intestinal paralysis and vascular complications (Fig. 2.B); and outcomes, including pancreatic-related infection, mortality within 14 days and total mortality (Fig. 2.C) among each platelet group. The incidence of renal and cardiovascular dysfunction was lower in the thrombocytosis and normal value groups than in the thrombocytopenia group. Mortality rates were similar. On the other hand, respiratory dysfunction, ANC, pancreatic necrosis and intestinal paralysis occurred at a higher rate in the thrombocytopenia group and the thrombocytosis group. This was also observed with regard to pancreatic-related infections APFC: Acute peripancreatic fluid collection, ANC: Acute necrotic collection Respiratory dysfunction was defined as PaO2/FiO2 less than 300 lasting more than 48 h; renal dysfunction was defined as creatinine more than 1.9 mg/dL; cardiovascular dysfunction was defined as nonresponsiveness to infusion, systolic blood pressure less than 90 mmHg, or the use of vasopressors; intestinal paralysis was defined as gastric elimination disorder and ileus, including intestinal necrosis and abdominal compartment syndrome; vascular complications were defined as the occurrence of pseudoaneurysm and venous thrombosis; and pancreatitis-related infection was defined as infected peripancreatic and/or pancreatic necrosis

Figure 2 shows the systemic complications, including respiratory, renal and cardiovascular dysfunction (Fig. 2.A); local complications, including APFC, ANC, pancreatic necrosis, intestinal paralysis and vascular complications (Fig. 2.B); and the outcome, including pancreatic-related infection, mortality within 14 days and total mortality (Fig. 2.C) among the three groups. The incidence of renal and cardiovascular dysfunction was lower in the thrombocytosis and normal value groups than in the thrombocytopenia group. This relationship was also observed in the mortality rate. However, respiratory dysfunction, ANC, pancreatic necrosis and intestinal paralysis occurred at a higher rate in the thrombocytopenia group and the thrombocytosis group than in normal value group. This relationship was also observed in pancreatic-related infections.

Regarding local complications and outcomes in patients with AP, multivariable logistic regression analysis showed that ANC, pancreatic necrosis and pancreatitis-related infection occurred at a significantly higher incidence in patients with thrombocytosis (ANC: OR, 7.360; 95% CI, 2.314–23.408; P = 0.001, pancreatic necrosis: OR, 3.735; 95% CI, 1.285–10.854; P = 0.015, pancreatic-related infection: OR, 9.815; 95% CI, 1.063–90.663; P = 0.044) (Table 4).

**Table 4:** Multivariable logistic regression analysis of predictive factors for the local complication or outcome in patients with AP.

**Variables**	Odds ratio	95% CI	p value
A. ANC			
APACHE II score	1.110	0.939–1.312	0.222
BISAP	1.293	0.674­ 2.479	0.439
Revised Atlanta classification: Severe	3.653	0.901­ 14.819	0.070
Platelet count groups			
Normal value (reference group)			
Thrombocytosis	7.360	2.314­ 23.408	0.001
Thrombocytopenia	5.273	1.373­ 20.249	0.015
B. Pancreatic necrosis			
APACHE II score	1.033	0.900- 1.185	0.646
BISAP	1.359	0.767­ 2.408	0.293
Revised Atlanta classification: Severe	0.658	0.174­ 2.496	0.539
Platelet count groups			
Normal value (reference group)			
Thrombocytosis	3.735	1.285­ 10.854	0.015
Thrombocytopenia	5.485	1.384­ 21.741	0.015
C. Pancreatitis related infection			
APACHE II score	1.059	0.903–1.242	0.479
BISAP	1.091	0.490­ 2.431	0.831
Revised Atlanta classification: Severe	2.388	0.541 10.540	0.251
Platelet count groups			
Normal value (reference group)			
Thrombocytosis	9.815	1.063­ 90.663	0.044
Thrombocytopenia	7.122	0.696­ 72.924	0.098

ANC: Acute necrotic collection, APACHE II: Acute Physiology and Chronic Health Evaluation II, BISAP: Bedside Index for Severity in Acute Pancreatitis

Pancreatitis-related infection was defined as peripancreatic infection and/or pancreatic necrosis

## Discussion

Until now, the relationship between AP, especially SAP, and platelets has been mostly reported in cases of thrombocytopenia [36, 37]. The reason is that an increased incidence of systemic complications and mortality have been reported [11, 38]. In contrast, thrombocytosis in AP has rarely been reported [23, 25]. In this study, patients with thrombocytopenia had more systemic complications and mortality, similar to previous reports. On the other hand, among the patients with thrombocytosis, 87.9% had SAP, and respiratory dysfunction, ANC, pancreatic necrosis and intestinal paralysis occurred at higher rates in the thrombocytosis and thrombocytopenia groups than in the normal value group. This relationship was also observed with pancreatic-related infections. Additionally, thrombocytosis was associated with a risk of ANC development (OR 7.360) and pancreatic necrosis appearance (OR 3.735) as local pancreatic complications and pancreatic-related infections (OR 9.815) as an outcome. The results of this study suggest that thrombocytosis in AP may indicate the occurrence of local pancreatic complications. Routine blood testing is a very simple and inexpensive approach and can be performed in almost all medical institutions. Therefore, these results may reduce the financial burden on patients and help to improve the ability of clinicians to manage patients with AP, especially SAP.

Evaluation of the systemic or local complications of AP is important, and the revised Atlanta Classification uses the modified Marshall scoring system [39] to evaluate organ failure for systemic complications and suggests that radiological criteria be used to evaluate local complications. Treatment strategies for infected ANC have been shown to improve the prognosis of late-stage AP [40]. ANC or intestinal paralysis is a known pancreatic complication that develops in necrotizing pancreatitis [35]. Furthermore, ANC is reported to occur in 20% of cases of AP, and 30% of those cases are reported to be infected [40]. Infected ANC is a cause of mortality in late-phase necrotizing pancreatitis [41]. In the present study, ANC and intestinal paralysis occurred with both thrombocytosis and thrombocytopenia, with a significantly higher incidence than that of the normal value group. This relationship was also similar for the outcome of the occurrence of pancreatic-related infections. In contrast, thrombocytosis was associated with less mortality than thrombocytopenia. Regarding this difference, previous studies have reported that infected necrosis without organ failure has a lower mortality rate than infected necrosis with persistent organ failure [42]. In this study, the thrombocytopenia group exhibited more severe cases based on the revised Atlanta classification, while the thrombocytosis group exhibited more moderate cases. Therefore, it is possible that the mortality rate was lower in the thrombocytosis group because the organ damage was transient.

Platelets, which provide the cellular link between the inflammatory response and the activation of coagulation, may play an important role in the initiation of AP and the development of serious complications [25]. MPV is an indicator of platelet activation and reflects the burden of inflammation [43]. A recently published systematic review also showed that MPV is lower at the onset than at remission of AP, independent of the severity of the disease [44]. This decrease in MPV is thought to reflect increased platelet consumption at the site of inflammation in the pancreas and distant organs [24]. Similar to previous reports, in the present study, MPV was lower than the reference value (10.2–13.2 fL) in all three groups; in particular, a significant decrease was observed in the thrombocytosis group. Additionally, sustained stimulation that activates platelets is known to cause a decrease in platelet size [45]. In this study, the peak platelet count in the thrombocytosis group occurred 12.22 ± 3.03 days after the onset of AP. Therefore, the low MPV with thrombocytosis suggests that the burden of platelet activation and inflammation may be sustained.

CRP, lymphocytes, and AT have been shown to be indicators suggestive of a systemic inflammatory response and have been shown to be variable in SAP [9, 11, 12]. LD has also been shown to be higher in severe cases than in moderate cases according to the revised Atlanta classification. If LD is not significantly increased, the probability of developing persistent organ failure is low [10, 46]. In this study, severe pancreatitis, with persistent organ damage, was more common with thrombocytopenia, and moderately severe acute pancreatitis, with only local complications and short-term organ damage, was more common with thrombocytosis according to the revised Atlanta classification. Additionally, in this study, differences in lymphocytes and CRP, LD, and AT levels were observed between patients with thrombocytosis and thrombocytopenia during hospitalization. The fact that these changes did not occur with thrombocytosis suggests that systemic inflammation may be slight compared to that with thrombocytopenia. These results suggest that the persistent inflammation seen with thrombocytosis may reflect local inflammation.

There are several limitations of this study. The major limitation is that this is a prospective observational study with a limited number of patients assessed at a single institution. This hospital is a regional core hospital and does not admit patients with acute pancreatitis intensively. The rate of severe pancreatitis was high due to the presence of many patients referred from other hospitals. Therefore, the proportion of mild AP in this study was small. Mild AP has a lower incidence of local or systemic complications and mortality than SAP [35], and if we had included more patients with mild pancreatitis, the incidence of complications and mortality might have been different. In addition, although this study included patients with AP within 48 h of onset, the course of the disease until hospitalization varied. Therefore, the date and time of platelet fluctuation may not be accurate because the date of blood collection was based on the date of admission, not the date of onset. The maturation time of platelets in healthy individuals is estimated to be approximately 5 days for megakaryocytes, and the platelet lifespan after release into peripheral blood is approximately 8 days [47]. On the other hand, under severe conditions, there is a decrease in platelet lifespan due to marked platelet consumption and the release of nonfunctional immature platelets into the peripheral blood [48]. In this study, the platelet count was assessed by instrumental measurement. Therefore, it is possible that platelets without platelet function were also measured. In addition, we did not measure the factors that evaluate platelet function and action. Therefore, we could not account for the mechanism of platelet function and the development of complications.

## Conclusions

Thrombocytosis occurring during the course of AP is often observed in severe pancreatitis and is associated with the development of ANC and pancreatic-related infections and the appearance of pancreatic necrosis. Thrombocytosis may be a predictor of the development of local complications.

## Data Availability

The datasets analyzed during the current study are not publicly available because of contracts with hospitals that provide data to the database but are available from the corresponding author on reasonable request.
